# 磁分散固相萃取-液相色谱-串联质谱法测定蜂蜜中38种抗生素残留

**DOI:** 10.3724/SP.J.1123.2025.07017

**Published:** 2026-04-08

**Authors:** Jing SUN, Yu ZHANG, Fengmao LIU, Wen ZHAO, Yue JIN, Lin ZHANG, Xiaofeng XUE, Rui CHEN, Huatian ZHOU

**Affiliations:** 1.中国农业科学院蜜蜂研究所，北京 100193; 1. Institute of Apiculture Research，Chinese Academy of Agricultural Sciences，Beijing 100193，China; 2.中国农业大学理学院，北京 100193; 2. College of Science，China Agricultural University，Beijing 100193，China; 3.知孔（上海）生物科技有限公司，上海 201818; 3. Zhikong Biotechnology Co. ，Ltd. ，Shanghai 201818，China; 4.北京联合大学，北京 100101; 4. College of Biochemical Engineering，Beijing Union University，Beijing 100101，China

**Keywords:** 磁分散固相萃取, 蜂蜜, 混合模式吸附材料, 抗生素残留, 绿色分析化学, magnetic dispersive solid-phase extraction （MDSPE）, honey, mixed-mode sorbent, antibiotic residues, green analytical chemistry

## Abstract

蜂蜜中抗生素残留的精准检测是保障蜂产品质量与规范用药监管的关键环节。针对蜂蜜中糖类含量高、黏度大、基质干扰强以及抗生素种类多样、残留标准覆盖有限等问题，本工作构建了一种基于混合模式亲水-疏水平衡（mHLB）磁性材料的磁分散固相萃取-高效液相色谱-串联质谱（MDSPE-HPLC-MS/MS）方法，用于蜂蜜中氯霉素、喹诺酮类和磺胺类共38种抗生素残留的定量分析。通过Plackett-Burman设计筛选影响mHLB萃取效率的关键因素，并结合响应面法系统优化MDSPE的前处理步骤，在确保分析性能的同时显著简化操作流程。在优化的MDSPE条件下（10 mg mHLB吸附5 mL样品提取液，1 mL甲醇洗脱），38种抗生素在0.02~200 μg/L范围内线性关系良好（*R*²>0.99），检出限为0.01~0.12 μg/kg，定量限为0.02~0.39 μg/kg，回收率为70.5%~109.3%，相对标准偏差低于10.8%。该方法可有效控制基质干扰，适用于痕量抗生素的准确定量。在42批次实际样品的应用中，检出部分禁用或尚未纳入限量目录的抗生素，提示蜂产品中潜在的用药风险与监管局限性。此外，与已有方法比较，本方法操作简便，灵敏度高，适用性强，绿色评分达0.73，具备良好的环境友好性和方法学先进性。本研究为蜂蜜中抗生素残留的风险监测和标准完善提供了可靠的技术支持，并可为复杂基质中痕量污染物的绿色分析方法的开发提供参考。

## Abstract (en)

Accurate antibiotic residue detection in honey is crucial for food safety and veterinary drug regulation in apiculture. Honey is a particularly challenging matrix. The high sugar content， strong viscosity， and pronounced matrix effects of the sample make it difficult to extract and detect target analytes effectively. Moreover， multiple antibiotics are used in beekeeping， while existing residue standards often provide incomplete coverage. These limitations highlight the urgent need for a robust and environmentally friendly multi-residue analytical method. In this study， a green and sensitive method was developed for the simultaneous determination of 38 antibiotic residues， including chloramphenicol， quinolones， and sulfonamides， in honey. Sample preparation was based on magnetic dispersive solid-phase extraction （MDSPE） using a mixed-mode hydrophilic-lipophilic balance （mHLB） magnetic sorbent. This sorbent combines hydrophilic and hydrophobic interactions， enabling efficient extraction of chemically diverse antibiotics. Method optimization was performed systematically. A Plackett-Burman design was used to screen seven factors influencing extraction. The sorbent dosage， sample volume， and extraction time were identified as the most significant. These variables were further optimized by response surface methodology， which minimized the number of experimental runs while clarifying interactions among factors. The final protocol required only 10 mg of sorbent， 5 mL of sample extract， and 1 mL of methanol for elution. This miniaturized approach represents an environmentally sustainable method. Chromatographic separation was achieved on a Poroshell 120 SB-C18 column （100 mm×2.1 mm，2.7 μm） using a gradient of acidified methanol and water. Detection was carried out by high performance liquid chromatography-tandem mass spectrometry （HPLC-MS/MS） with electrospray ionization operated in both positive and negative modes， and multiple reaction monitoring ensured high sensitivity and selectivity. The method was fully validated. Excellent linearity was obtained for all analytes in the range of 0.02-200 μg/L， with correlation coefficients above 0.99. Limits of detection ranged from 0.01 to 0.12 μg/kg， while limits of quantification ranged from 0.02 to 0.39 μg/kg. Recoveries at three spiked levels ranged from 70.5% to 109.3%， with relative standard deviations （RSDs） below 10.8%. Matrix effects ranged from 0.82 to 1.19， indicating effective suppression of interference and reliable quantification in complex honey samples. The method was successfully applied to 42 commercial honey batches from different geographical regions. Several antibiotics were detected， including chloramphenicol， as well as other compounds without established maximum residue limits. These findings indicate potential misuse of antibiotics in beekeeping and reveal important gaps in current regulatory frameworks， underscoring the need for continuous monitoring and timely updates to residue standards. In addition to high sensitivity and wide applicability， the method demonstrated strong environmental advantages. Green performance was assessed using the AGREEprep tool， which evaluates twelve principles of sustainable sample preparation. The method achieved a high score of 0.73， reflecting minimal solvent consumption， low sorbent use， low energy demand， and negligible hazardous waste. These features align with the principles of green analytical chemistry and significantly reduce the environmental impact of residue analysis. In conclusion， this work establishes an MDSPE-HPLC-MS/MS for multi-class antibiotic residue detection in honey. The method combines simplicity， sensitivity， robustness， and environmental compatibility. It provides reliable technical support for risk assessment and regulatory improvement of antibiotic residues in honey， and it offers a valuable reference for the development of green analytical methods for trace contaminants in other complex food and environmental matrices.

蜂蜜是由蜜蜂采集植物花蜜酿造而成的天然产品，富含糖类、氨基酸、蛋白质以及生物活性物质，具有独特的营养和药用价值^［1］^。然而，在蜜蜂养殖过程中，蜂群易受到致病菌侵袭及寄生虫感染^［2］^，为控制蜂病、提高产量，常使用抗生素^［3］^如氯霉素类（chloramphenicols， CAPs）、磺胺类（sulfonamides， SAs）、喹诺酮类（quinolones， QNs）等药物进行干预^［4］^。在实际生产中，由于用药剂量不当、停药期不足及监管薄弱等原因，蜂蜜中极易造成抗生素残留，进而对食品安全和消费者健康构成威胁^［5］^。针对常见的抗生素残留，我国已出台一系列监管措施^［6，7］^。而随着蜂药使用类型日益复杂，实际检出的抗生素种类远超现行标准覆盖范围，亟需构建覆盖广、灵敏度高、适用于不同蜂蜜基质的多残留检测技术体系，以提升风险识别能力，为标准修订提供依据。

蜂蜜样品具有高糖、高黏度和基质复杂等特点，使得痕量抗生素的提取与净化极具挑战性。传统样品前处理技术如液-液萃取（LLE）^［8］^、固相萃取（SPE）^［9］^、QuEChERS^［10］^等，虽然被广泛应用于食品中抗生素残留分析，但其处理流程烦琐，选择性不足，有机溶剂用量大，难以满足现代蜂产品监管对高通量、低检出限和绿色环保的分析需求。磁分散固相萃取（magnetic dispersive solid-phase extraction， MDSPE）作为近年来发展迅速的新型样品前处理技术^［11］^，通过功能化磁性材料对复杂基质中目标物的快速吸附，并借助外加磁场实现高效分离，省去传统SPE中的离心、过滤等步骤，显著提高前处理效率^［12，13］^。随着材料科学的发展，越来越多结构可调的磁性吸附剂被用于MDSPE中^［14，15］^，不仅可以实现特定目标物的选择性吸附^［16， 17］^，在复杂基质的多残留检测中也表现出显著优势^［18］^。其中基于混合模式亲水-疏水平衡（mixed-mode hydrophilic-lipophilic balance， mHLB）开发的磁性材料，凭借其同时具备疏水作用、氢键结合与离子交换能力，可广泛适用于结构差异显著的目标物的共提取，在多残留分析中展现出良好的应用潜力^［19］^，但尚未见其在高糖蜂蜜基质中的相关应用研究。

在绿色分析化学（green analytical chemistry， GAC）理念的推动下，样品前处理技术的环境友好性和可持续性正逐步成为评价方法先进性的重要指标^［20］^。理想的绿色前处理方法不仅应满足高灵敏度与高选择性，还应兼顾试剂消耗少、步骤简化、时间成本低等目标。近年来，Plackett-Burman设计被广泛应用于前处理参数的快速筛选^［21］^，可有效识别影响因素并缩小优化范围；结合响应面分析方法，可系统评估变量间的交互作用，在较少实验次数下实现条件最优化^［22］^。同时，绿色评估工具如Analytical GREEnness Calculator（AGREE）^［23］^等为分析流程的环境影响提供可量化、可视化的评价依据，逐渐成为绿色方法评价的重要手段。这些技术的整合有望为高效、绿色、智能化的多残留分析方法的构建提供实践路径。

本研究基于mHLB磁性材料建立了一种磁分散固相萃取-高效液相色谱-串联质谱法（MDSPE-HPLC-MS/MS），以实现蜂蜜中氯霉素类、磺胺类与喹诺酮类等38种抗生素的高效准确定量。通过Plackett-Burman设计筛选关键参数，并结合响应面优化前处理条件，实现灵敏度高、检出限低、操作简便与绿色低耗的有机统一。并对全国42批次不同蜜源的蜂蜜样品进行抗生素残留监测，为蜂产品中抗生素残留的风险筛查、标准完善和风险监管提供技术支撑。

## 1 实验部分

### 1.1 仪器、试剂与材料

Agilent 1290Ⅱ-6495高效液相色谱-串联质谱仪（美国Agilent公司），多管涡旋混匀仪（北京兰杰柯科技有限公司）。

标准物质：氯霉素、19种喹诺酮类和18种磺胺类药物的标准品均购于天津阿尔塔科技有限公司，纯度均大于90%。将上述标准物质用甲醇配制成质量浓度均为100 mg/L的混合标准溶液，冷冻保存备用。

试剂与样品：甲醇、乙腈（色谱纯，美国Fisher公司）；甲酸（色谱纯，上海安谱公司）；甲酸铵（优级纯，上海阿拉丁公司）；mHLB磁分散固相萃取材料（艾捷博雅科技有限公司）；实验用水为超纯水（上海乐枫生物科技有限公司）；在国内10家加工企业、9家合作社和4家蜂场中随机抽取百花、洋槐、枣花、荆条和油菜等42份蜂蜜样品。

### 1.2 样品前处理

准确称取蜂蜜样品1.00 g置于10 mL离心管中，加入5 mL 0.1 mol/L乙酸铵水溶液，涡旋1 min充分混匀，获得样品提取液。

取10.0 mg mHLB磁性固相萃取材料置于15 mL离心管中，依次用1.0 mL甲醇和1.0 mL超纯水，涡旋30 s活化与平衡，磁分离后弃去上清液。向离心管加入样品提取液，涡旋1 min弃去上清液；向离心管中加入2 mL纯水淋洗，涡旋30 s，弃去上清液；加入1 mL甲醇洗脱，涡旋1 min，收集洗脱液，过0.22 μm微孔尼龙滤膜，用超纯水稀释5倍后待测。整体前处理过程可在10 min内完成，操作流程见图1。

**图1:**
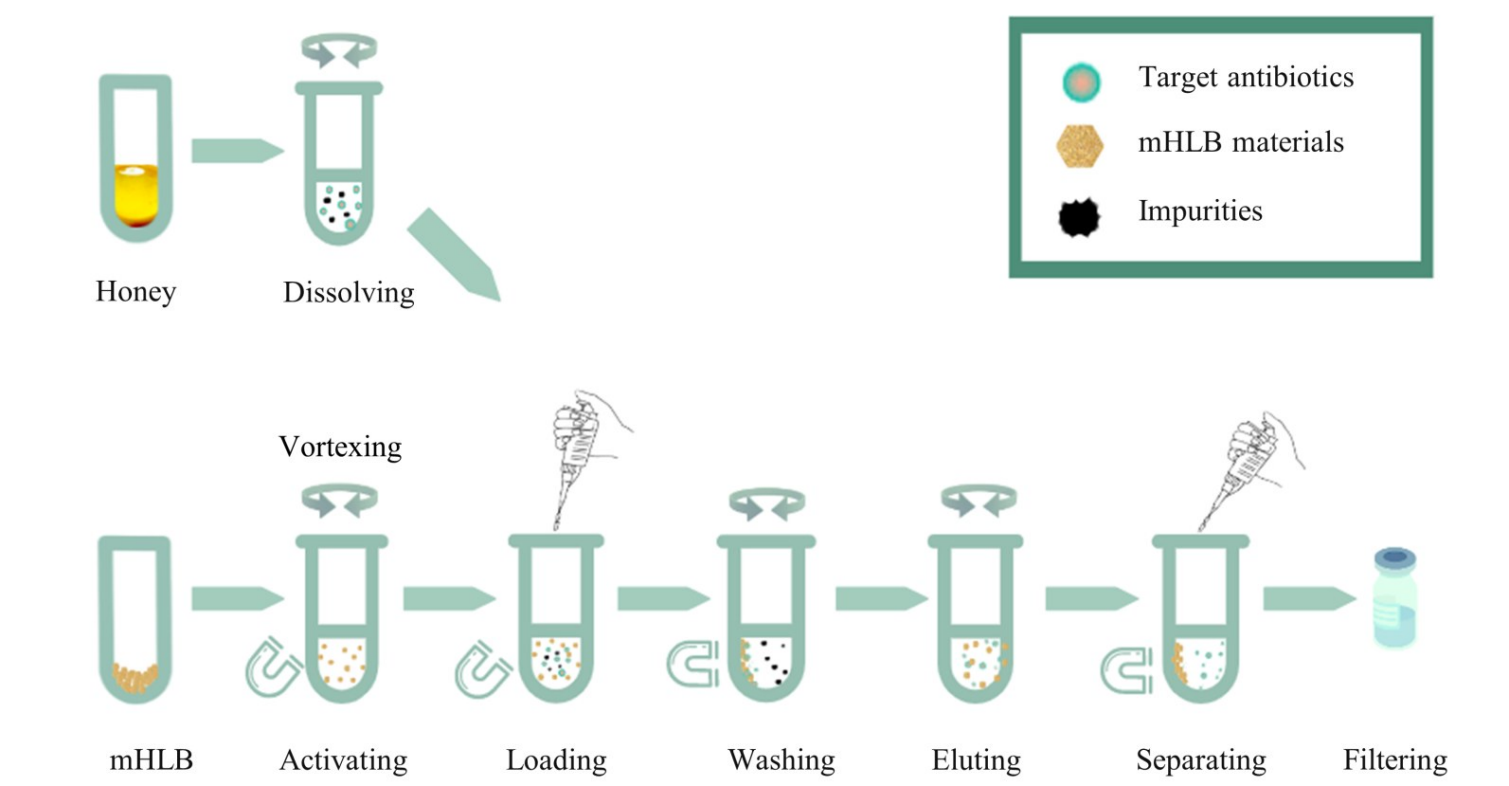
磁分散固相萃取的操作流程

### 1.3 分析条件

#### 1.3.1 色谱条件

Poroshell 120 SB-C_18_色谱柱（100 mm×2.1 mm，2.7 μm）；柱温为30 ℃；进样体积为5 μL；流动相A为含2 mmol/L甲酸铵的0.1%甲酸水溶液，流动相B为甲醇；洗脱程序：0~1.5 min，5%B；1.5~5.0 min，5%B~25%B；5.0~8.0 min，25%B~35%B；8.0~10.0 min，35%B~65%B；10.0~12.0 min，65%B~85%B；12.0~13.0 min，85%B；13.0~13.1 min，85%B~5%B；13.1~16.0 min，5%B。流速为0.3 mL/min。

#### 1.3.2 质谱条件

电喷雾离子源（ESI），氯霉素（LV）为负离子模式（ESI^-^），其余化合物均为正离子模式（ESI^+^）；多反应监测模式（MRM）；离子源温度350 ℃。38种抗生素的其他质谱参数见表1。

**表1:** 38种抗生素的质谱参数

Compound	Abbreviation	CAS No.	Retention time/min	Parent ion （*m/z*）	Product ions （*m/z*）	CEs/eV
Chloramphenicol compounds （氯霉素类化合物）	CAPs					
Chloramphenicol（氯霉素）	LV	56-75-7	8.76	320.9	257.0^*^， 194.0	8， 9
Quinolone compounds （喹诺酮类化合物）	QNs					
Difloxacin （双氟沙星）	DIF	98106-17-3	8.78	400.0	356.1^*^， 382.1	20， 20
Lomefloxacin （洛美沙星）	LOM	98079-51-7	8.33	352.0	265.0^*^， 334.0	24， 22
Danofloxacin （达氟沙星）	DAN	112398-08-0	8.26	358.2	340.1^*^， 255.0	25， 46
Enrofloxacin （恩诺沙星）	ENR	93106-60-6	8.17	360.0	316.2^*^， 342.1	20， 20
Orbifloxacin （奥比杀星）	ORB	113617-63-3	8.51	396.2	295.1^*^， 352.1	22， 15
Gatifloxacin （加替沙星）	GAT	112811-59-3	9.43	376.0	332.0^*^， 261.0	15， 24
Marbofloxacin （马波杀星）	MAR	115550-35-1	6.87	363.5	345.1^*^， 320.1	20， 10
Enoxacin （依诺沙星）	ENO	74011-58-8	7.46	321.0	303.1^*^， 232.0	18， 38
Fleroxacin （氟罗杀星）	FLE	79660-72-3	6.98	370.1	326.0^*^， 269.0	18， 28
Norfloxacin （诺氟沙星）	NOR	70458-96-7	7.67	320.0	302.1^*^， 276.1	20， 15
Ofloxacin （氧氟沙星）	OFL	82419-36-1	7.40	362.0	261.1^*^， 318.1	26， 15
Pefloxacin （培氟沙星）	PEF	70458-92-3	7.45	334.0	316.0^*^， 290.0	25， 20
Ciprofloxacin （环丙沙星）	CIP	85721-33-1	7.97	332.1	314.1^*^， 288.0	20， 24
Sparfloxacin （司帕沙星）	SPA	110871-86-8	9.89	393.1	349.0^*^， 292.0	21， 36
Cinoxacin （西诺杀星）	CIN	28657-80-9	9.88	263.0	189.0^*^， 217.0	30， 20
Oxolinic acid （噁喹酸）	OXO	14698-29-4	10.31	262.1	244.2^*^， 216.0	16， 15
Nalidixic acid （萘啶酸）	NAL	389-08-2	10.99	233.0	215.0^*^， 187.0	10， 20
Flumequine （氟甲喹）	FLU	42835-25-6	10.10	262.1	244.1^*^， 202.1	12， 18
Sarafloxacin （沙拉沙星）	SAR	98105-99-8	9.13	386.1	368.1^*^， 342.1	20， 15
Sulfonamide compounds （磺胺类化合物）	SAs					
Sulfadiazine （磺胺嘧啶）	SDZ	68-35-9	4.67	251.0	156.0^*^， 108.0	10， 22
Sulfathiazole （磺胺噻唑）	STZ	72-14-0	5.25	256.0	156.0^*^， 108.0	10， 25
Sulfapyridine （磺胺吡啶）	SPD	144-83-2	5.36	250.0	156.0^*^， 184.0	15， 15
Sulfamerazine （磺胺甲基嘧啶）	SMR	127-79-7	5.72	265.0	156.0^*^， 172.0	15， 15
Sulfameter （磺胺对甲氧嘧啶）	SME	651-06-9	6.90	281.0	156.0^*^， 92.0	10， 20
Sulfamethizole （磺胺甲噻二唑）	SMI	144-82-1	6.61	271.0	156.0^*^， 108.0	10， 25
Sulfamethazine （磺胺二甲基嘧啶）	SMA	57-68-1	6.62	279.0	186.1^*^， 156.0	15， 10
Sulfamethoxypyridazine （磺胺甲氧哒嗪）	SMP	80-35-3	6.45	281.0	156.0^*^， 92.0	10， 20
Sulfachloropyridazine （磺胺氯哒嗪）	SCP	80-32-0	7.18	285.0	156.0^*^， 108.0	15， 20
Sulfamonomethoxine （磺胺间甲氧嘧啶）	SMM	1220-83-3	7.65	281.0	156.0^*^， 92.0	15， 15
Sulfadimethoxazole （磺胺二甲异噁唑）	SSZ	127-69-5	7.97	268.0	113.0^*^， 156.0	10， 5
Sulfadoxine （磺胺邻二甲氧嘧啶）	SDX	2447-57-6	7.93	311.0	156.0^*^， 108.0	15， 20
Sulfamethoxazole （磺胺甲噁唑）	SMX	723-46-6	7.36	254.0	156.0^*^， 147.0	15， 15
Benzenesulfonamide （磺胺苯酰）	SB	98-10-2	8.50	277.1	156.0^*^， 108.0	6， 22
Sulfaphenazole （磺胺苯吡唑）	SPZ	526-08-9	9.24	315.0	160.0^*^， 156.0	20， 20
Sulfaclozina （磺胺氯吡嗪）	SCZ	102-65-8	7.18	285.0	156.0^*^， 108.0	10， 22
Sulfadimethoxine （磺胺间二甲氧嘧啶）	SDM	155-91-9	7.93	311.0	156.0^*^， 218.0	15， 20
Sulfaquinoxaline （磺胺喹噁啉）	SQX	59-40-5	10.13	301.1	92.0^*^， 156.0	29， 11

* Quantitative ion.

### 1.4 实验设计

#### 1.4.1 Plackett-Burman实验设计

通过Mintab Statistical Software 22软件进行Plackett-Burman实验设计，以材料用量（*X*_1_）、上样体积（*X*_2_）、上样时间（*X*_3_）、淋洗体积（*X*_4_）、洗脱体积（*X*_5_）、洗脱时间（*X*_6_）*、*洗脱剂比例（甲醇的体积分数，*X*_7_）等7个因素为研究对象，在+1和-1两水平下进行Plackett-Burman实验设计，筛选影响蜂蜜中38种抗生素提取回收率的显著性因素。

#### 1.4.2 响应面优化设计

通过Design-Expert 13.0软件，基于Plackett-Burman实验筛选的关键因素（洗脱剂比例、洗脱体积、材料用量、上样体积），在-1、0和+1 3个水平下进行Box-Behnken设计（BBD）。以回收率为响应值，构建二次多项式回归模型，并利用三维响应面图解析因素交互作用，确定最优参数组合。

### 1.5 绿色评估

采用AGREEprep工具，对本研究建立的MDSPE-HPLC-MS/MS方法进行系统性绿色度评估，同时选取文献报道的典型前处理方法作为对比。基于绿色分析化学的12项核心原则，从以下4个维度进行评估：原位分析可行性（1）、样品用量（2）、设备需求（3）等分析过程特性；步骤数量（4）、自动化程度（5）、衍生化需求（6）等操作流程特征；废弃物产生量（7）、通量（8）、能耗（9）等环境负荷指标；试剂可再生性（10）、毒性试剂使用（11）、操作风险（12）等安全性能。结果以象形图显示，其中深红色代表低分，深绿色代表高分，分数越接近1，表明该评估项在绿色分析化学标准下的表现越优，绿色程度越高。

## 2 结果与讨论

### 2.1 提取方法优化结果

#### 2.1.1 Plackett-Burman实验结果分析

Plackett-Burman实验设计过程及回收率结果见表2；对回收率实验结果进行方差分析，结果见表3。以LV分析结果为例，方差分析模型的回归方程为*Y=*67.9*-*0.033*X*_1_ *-*0.34*X*_2_ *+*1.68*X*_3_ *+*0.55*X*_4_ *-*9.88*X*_5_ *+*7.63*X*_6_ *+*68.11*X*_7_，该模型的*P*值=0.006<0.01，说明该数据模型显著可靠，决定系数（*R* ^2^）为0.972 1，可以有效预测蜂蜜中氯霉素的提取回收率。进一步分析3个模型的*F*值发现，影响蜂蜜中3类抗生素的提取回收率的关键因素依次为*X*_7_（洗脱剂比例）>*X*_5_（洗脱体积）>*X*_1_（材料用量）>*X*_2_（上样体积）>*X*_3_（上样时间）>*X*_4_（淋洗体积）>*X*_6_（洗脱时间）。

**表2:** Plackett-Burman实验设计及结果

Running sequence	*X*_1_ */*mg	*X*_2_ */*mL	*X*_3_ */*min	*X*_4_ */*mL	*X*_5_ */*mL	*X*_6_ */*min	*X*_7_ */*%	Recoveries/%		
								LV	QNs	SAs
1	5.0	1.0	0.5	1.0	0.5	0.5	20	1.9	6.7	5.0
2	5.0	1.0	0.5	3.0	2.0	0.5	100	118.8	87.2	75.5
3	30.0	5.0	0.5	3.0	2.0	0.5	100	123.1	88.9	80.4
4	5.0	5.0	0.5	1.0	0.5	3.0	100	169.7	80.1	67.4
5	30.0	1.0	0.5	1.0	2.0	3.0	20	2.0	6.7	6.3
6	30.0	5.0	0.5	3.0	0.5	3.0	20	0.0	0.4	0.2
7	30.0	5.0	3.0	1.0	2.0	0.5	20	2.0	9.6	4.3
8	30.0	1.0	3.0	1.0	0.5	0.5	100	122.5	39.6	50.8
9	5.0	5.0	3.0	3.0	0.5	0.5	20	2.4	9.6	5.3
10	5.0	1.0	3.0	3.0	2.0	3.0	20	11.0	19.5	21.9
11	5.0	5.0	3.0	1.0	2.0	3.0	100	126.7	85.6	78.3
12	30.0	1.0	3.0	3.0	0.5	3.0	100	175.9	49.0	62.8

*X*_1_： material consumption； *X*_2_： loading volume； *X*_3_： loading time； *X*_4_： wash volume； *X*_5_： eluent volume； *X*_6_： eluent time； *X*_7_： volume percentage of methanol.

**表3:** Plackett-Burman实验方差分析结果

Source	CAP			QNs			SAs		
	*F*-value	*P*-value	Significance	*F*-value	*P*-value	Significance	*F*-value	*P*-value	Significance
Model	19.93	0.006	**	131.56	0.000	**	69.20	0.001	**
Linear	19.93	0.006	**	131.56	0.000	**	69.20	0.001	**
*X*_1_	0.00	0.948	—	47.80	0.002	**	7.69	0.050	*
*X*_2_	0.01	0.913	—	22.85	0.009	**	0.62	0.476	—
*X*_3_	0.13	0.739	—	17.30	0.014	*	0.41	0.557	—
*X*_4_	0.01	0.930	—	3.71	0.126	—	3.79	0.123	—
*X*_5_	1.60	0.275	—	66.92	0.001	**	18.46	0.013	*
*X*_6_	2.65	0.179	—	0.00	0.984	—	0.79	0.425	—
*X*_7_	135.07	0.000	**	762.34	0.000	**	452.62	0.000	**
*R* ^2^	0.9721 0.9233			0.9957 0.9881			0.9818 0.9775		
Adj. *R* ^2^									

** extremely significant； * significant； —： not significant.

此外，通过回收率的方差分析结果，获得标准化效应的Pareto图，如图2所示。标准化效应是估计效应除以其标准误差，即每个效应的*t*统计量。其条形的长度与标准化效应成正比，*α*>0.05说明对应因子在95%置信水平下具有统计显著性。结合3个模型标准化效应的Pareto图发现，洗脱剂比例对于3类抗生素的提取回收率影响均极为显著，洗脱体积、材料用量对于喹诺酮和磺胺类抗生素的提取率也呈现不同程度的显著影响。综上，以洗脱剂比例、洗脱体积、材料用量和上样体积4个因素为影响38种抗生素回收率的关键因素，进一步进行响应面优化设计。综合考虑时间和成本，后续实验将上样时间和洗脱时间固定为1 min，淋洗体积为2 mL。

**图2:**
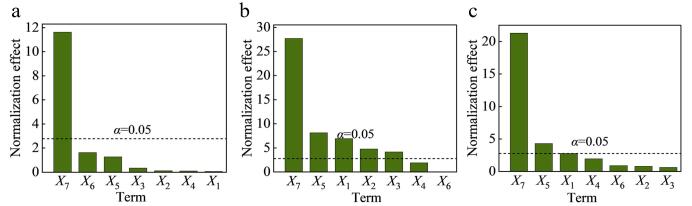
（a）氯霉素、（b）喹诺酮类和（c）磺胺类标准化效应的Pareto图

#### 2.1.2 响应面优化实验结果

响应面实验设计与结果如表4所示。3类抗生素的平均提取回收率（*Y*）与材料用量*X*_1_、上样体积*X*_2_、洗脱体积*X*_5_、洗脱剂比例*X*_7_ 4个因素的多元回归方程见式（1）。


*Y=*46-7.48*X*_1_-0.4159*X*_2_+6.55*X*_5_+25.66*X*_7_+5.4*X*_1_
*X*_2_+4.55*X*_5_
*X*_1_-3.51*X*_5_
*X*_2_+1.09*X*_7_
*X*_1_-2.77*X*_7_
*X*_2_-5.05*X*_7_
*X*_5_-3.43*X*_1_^2^+8.51*X*_2_^2^+4.59*X*_5_^2^-14.68*X*_7_^2^ （1）

**表4:** 响应面优化实验设计

Running sequence	*X*_1_/mg	*X*_2_/mL	*X*_5_/mL	*X*_7_/%	Recovery/%
1	17.5	3	0.5	20	3.3
2	17.5	3	0.5	100	67.2
3	17.5	3	2	20	12.6
4	17.5	3	2	100	56.3
5	5	1	1.25	60	64.7
6	30	1	1.25	60	38.4
7	5	5	1.25	60	50.8
8	30	5	1.25	60	46.2
9	17.5	1	1.25	20	9.1
10	17.5	1	1.25	100	80.6
11	17.5	5	1.25	20	8.0
12	17.5	5	1.25	100	68.4
13	5	3	0.5	60	59.7
14	5	3	2	60	59.5
15	30	3	0.5	60	29.0
16	30	3	2	60	47.1
17	5	3	1.25	20	15.2
18	5	3	1.25	100	47.2
19	30	3	1.25	20	5.1
20	30	3	1.25	100	41.6
21	17.5	1	0.5	60	35.8
22	17.5	1	2	60	74.0
23	17.5	5	0.5	60	50.0
24	17.5	5	2	60	74.2
25	17.5	3	1.25	60	47.6
26	17.5	3	1.25	60	51.9
27	17.5	3	1.25	60	38.5

实验结果的方差分析如表5所示。该模型的*F*值为7.5，*P*值=0.000 6<0.01，说明多元回归模型极为显著，失拟项>0.05，显示不显著，说明回归方程拟合效果较好。*R* ^2^=0.897 4，该模型可以解释89.74%的实验结果，校正决定系数（Adj. *R* ^2^）用来说明用自变量解释因变量变异的程度，Adj. *R* ^2^=0.777 7，可见该模型可以解释77.77%的变异。分析标准化残差概率图，如图3所示，该模型的残差概率均匀分布在一条直线上，所有样品的误差没有异常点，说明该模型的预测准确性较好，可以用于进一步分析。

**表5:** 响应面优化实验方差分析结果

Source	Sum of squares	df	Mean square	*F-*value	*P-*value	Significance
Model	12007.74	14	857.7	7.5	0.0006	**
*X*_1_	672.19	1	672.19	5.88	0.0321	*
*X*_2_	2.08	1	2.08	0.0181	0.8951	—
*X*_5_	515.47	1	515.47	4.51	0.0453	*
*X*_7_	7898.35	1	7 898.35	69.04	<0.0001	**
*X*_7_ *X*_5_	101.87	1	101.87	0.8904	0.0364	*
*X*_7_ *X*_1_	4.78	1	4.78	0.0417	0.8415	—
*X*_7_ *X*_2_	30.59	1	30.59	0.2674	0.6145	—
*X*_5_ *X*_1_	82.73	1	82.73	0.7231	0.4118	—
*X*_5_ *X*_2_	49.36	1	49.36	0.4315	0.0507	—
*X*_1_ *X*_2_	116.65	1	116.65	1.02	0.3325	—
*X*_1_²	62.74	1	62.74	0.5484	0.4732	—
*X*_2_²	386.67	1	386.67	3.38	0.0099	**
*X*_5_²	112.53	1	112.53	0.9836	0.3409	—
*X*_7_²	1149.42	1	1 149.42	10.05	0.0081	**
Residual	1372.86	12	114.4			
Lack of fit	1280.24	10	128.02	2.76	0.2948	—
Pure error	92.62	2	46.31			
Cor total	13380.6	26				
*R* ^2^ Adj. *R* ^2^	0.8974 0.7777					

** extremely significant； * significant； —： not significant.

**图3:**
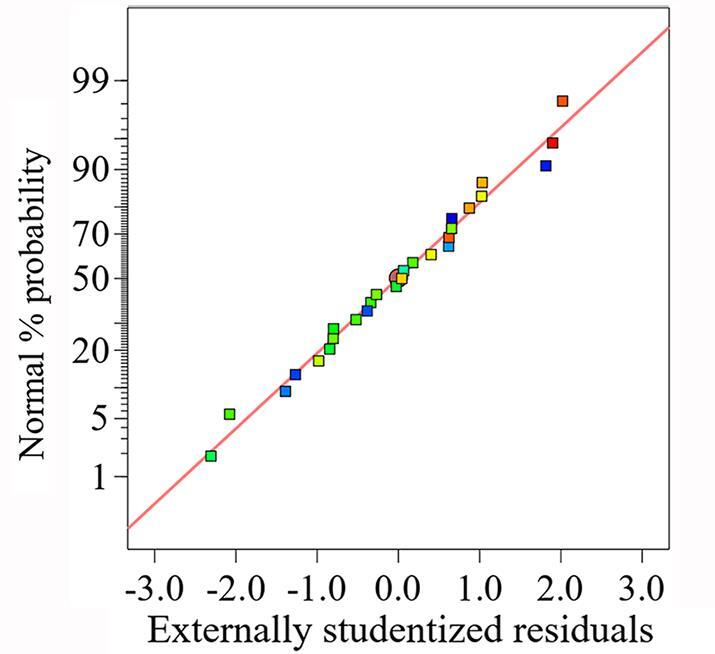
响应面优化实验方差分析的标准化残差概率图

根据*F*值进一步推断，关键因素的显著性影响能力依次为洗脱剂比例>材料用量>洗脱体积>上样体积，回归模型中的一次项*X*_1_、*X*_5_、*X*_7_，其中*X*_7_-洗脱剂比例的影响极为显著，二次项*X*_2_^2^、*X*_7_^2^，交互项的*X*_2_ *X*_5_、*X*_5_ *X*_7_影响显著。

由于洗脱剂比例*X*_7_对回收率的影响最为显著，因此进一步分析*X*_7_ *X*_1_、*X*_7_ *X*_2_、*X*_7_ *X*_5_响应曲面图，如图4所示，洗脱剂比例越高，回收率越高；当洗脱剂比例为100%甲醇时，洗脱体积对于回收率的总体影响不明显；材料用量在5~20 mg时，回收率均表现较高水平；上样体积接近1 mL或5 mL时回收率更高。综合Design-Expert推荐优化的结果、实际操作和成本，确定优化条件为材料用量10 mg，上样体积5 mL，洗脱剂比例100%甲醇，洗脱体积1 mL。

**图4:**
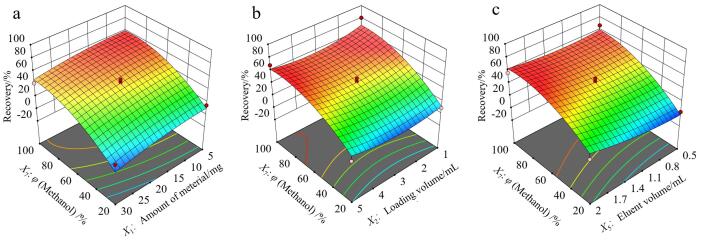
（a）*X*_7_ *X*_1_、（b）*X*_7_ *X*_2_、（c）*X*_7_ *X*_5_的响应曲面图

### 2.2 方法学验证

#### 2.2.1 线性关系、灵敏度及基质效应评价

以响应面优化筛选出的条件进行样品前处理的方法学验证，包括方法的线性关系、检出限（limit of detection， LOD）、定量限（limit of quantification， LOQ）、基质效应（matrix effect， ME）、回收率和精密度，结果如表6所示。氯霉素、喹诺酮类和磺胺类药物分别在0.02~100 μg/L、0.1~100 μg/L、0.1~200 μg/L范围内具有良好的线性关系，*R* ^2^均大于0.99。以信噪比（*S*/*N*）为3和10分别确定LOD和LOQ，其中氯霉素的LOD为0.01 μg/kg，LOQ为0.03 μg/kg；喹诺酮类抗生素的LOD为0.01~0.03 μg/kg，LOQ为0.02~0.1 μg/kg；磺胺类抗生素的LOD为0.02~0.03 μg/kg，LOQ为0.04~0.1 μg/kg。38种抗生素混合标准溶液的HPLC-MS/MS总离子流图见图5。

**表6:** 蜂蜜中38种抗生素的线性关系、检出限、定量限以及不同水平下的回收率和RSD（*n*=3）

Compound	Linear range/（μg/L）	*R* ^2^	LOD/ （μg/kg）	LOQ/ （μg/kg）	1 μg/kg		10 μg/kg		100 μg/kg	
					Recovery/%	RSD/%	Recovery/%	RSD/%	Recovery/%	RSD/%
CAPs										
LV	0.02-100	0.9969	0.01	0.03	85.2	8.3	82.4	6.9	88.7	5.2
QNs										
DIF	0.1-100	0.9982	0.01	0.03	75.2	6.3	73.1	9.9	73.2	7.5
LOM	0.1-100	0.9952	0.01	0.02	71.2	2.4	82.6	5.8	98.0	10.8
DAN	0.1-100	0.9944	0.01	0.06	84.9	9.1	109.3	8.2	105.8	7.2
ENR	0.1-100	0.9994	0.01	0.02	84.4	7.6	86.7	5.3	91.6	5.8
ORB	0.1-100	0.9908	0.01	0.02	74.9	2.2	79.2	7.0	84.7	4.7
GAT	0.1-100	0.9960	0.01	0.02	70.4	3.7	81.1	8.5	86.3	2.3
MAR	0.1-100	0.9963	0.01	0.02	74.4	3.3	86.8	2.3	85.1	4.3
ENO	0.1-100	0.9949	0.01	0.03	71.7	3.4	98.8	4.8	100.7	4.9
FLE	0.1-100	0.9966	0.01	0.02	79.0	0.7	86.7	2.4	92.8	7.5
NOR	0.1-100	1.0000	0.03	0.08	72.8	4.0	101.3	3.8	103.9	4.6
OFL	0.1-100	0.9990	0.01	0.02	83.0	9.8	87.7	2.5	94.5	6.5
PEF	0.1-100	0.9992	0.01	0.04	103.7	5.4	102.0	5.6	101.6	6.2
CIP	0.1-100	1.0000	0.01	0.03	70.7	4.3	85.9	4.6	100.6	9.9
SPA	0.1-100	0.9993	0.01	0.02	73.2	7.5	81.3	5.9	91.8	2.4
CIN	0.1-100	0.9999	0.02	0.04	70.9	6.3	71.6	3.8	72.9	3.7
OXO	0.1-100	0.9913	0.01	0.03	72.0	8.9	73.8	1.4	91.3	7.9
NAL	0.1-100	0.9932	0.01	0.02	72.5	0.4	75.6	4.5	80.7	0.1
FLU	0.1-100	0.9914	0.01	0.03	70.5	9.2	77.3	0.9	86.2	1.6
SAR	0.1-100	0.9998	0.01	0.02	71.8	7.7	85.2	7.4	85.4	8.1
SAs										
SDZ	0.1-200	1.0000	0.04	0.12	70.8	4.3	72.9	2.5	77.7	1.3
STZ	0.1-200	0.9979	0.02	0.04	71.1	2.8	80.4	0.8	79.8	0.7
SPD	0.1-200	0.9997	0.01	0.03	71.4	5.8	73.3	0.8	75.6	2.8
SMR	0.1-200	1.0000	0.02	0.06	75.6	2.8	73.8	1.2	84.7	0.8
SME	0.1-200	1.0000	0.01	0.04	72.7	3.2	76.0	2.5	82.6	1.7
SMI	0.1-200	0.9996	0.03	0.08	71.3	3.6	72.7	3.2	76.6	2.2
SMA	0.1-200	1.0000	0.02	0.05	75.4	4.3	76.8	0.8	81.9	1.2
SMP	0.1-200	0.9998	0.02	0.04	72.6	5.8	71.8	2.9	81.7	1.7
SCP	0.1-200	0.9977	0.03	0.09	72.2	4.0	74.1	1.8	82.0	0.9
SMM	0.1-200	0.9983	0.03	0.09	71.7	4.1	74.4	5.5	84.2	1.7
SSZ	0.1-200	0.9998	0.10	0.33	75.7	1.1	77.6	1.2	77.6	1.2
SDX	0.1-200	0.9999	0.02	0.06	71.7	2.7	74.2	0.8	82.2	0.3
SMX	0.1-200	0.9998	0.03	0.08	72.5	4.6	74.3	3.0	80.7	0.7
SB	0.1-200	1.0000	0.02	0.07	76.5	3.1	71.4	1.6	78.6	1.2
SPZ	0.1-200	1.0000	0.03	0.1	75.9	5.3	72.1	1.9	83.2	0.9
SCZ	0.1-200	0.9998	0.12	0.39	72.0	2.2	71.6	0.3	83.1	1.7
SDM	0.1-200	1.0000	0.02	0.05	70.5	1.2	70.5	1.2	83.4	0.4
SQX	0.1-200	0.9998	0.02	0.06	81.5	1.5	72.5	0.9	78.5	2.5

**图5:**
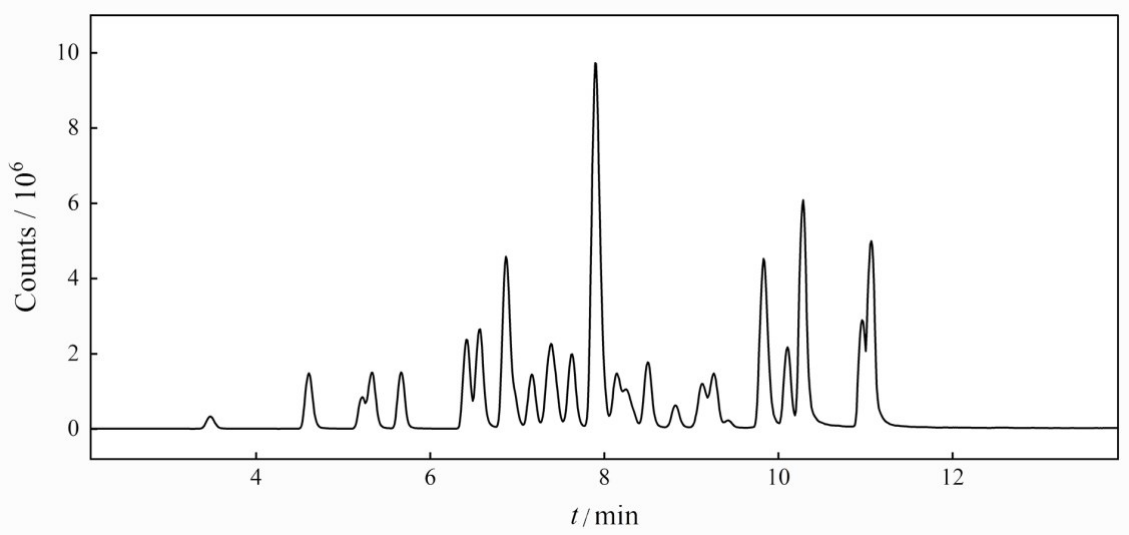
蜂蜜样品中氯霉素、喹诺酮类和磺胺类38种抗生素的总离子色谱图

根据基质标准曲线与溶剂标准曲线的斜率比评价基质效应，如附表1（www.chrom-China.com）所示。当ME>1时，表明存在基质增强效应；当ME<1时，表明存在基质抑制效应；当0.8<ME<1.2时，基质效应影响较小，可忽略。经MDSPE净化后，38种抗生素的基质效应为0.82~1.19（图6），为可忽略的基质效应，表明所建立的MDSPE方法能有效消除蜂蜜复杂基质的干扰。

**图6:**
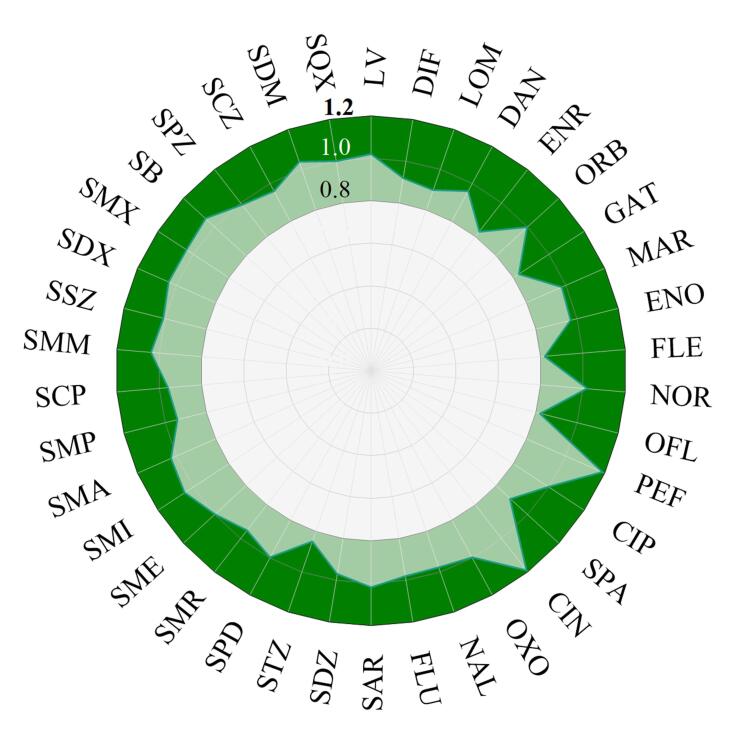
蜂蜜中38种抗生素的基质效应

#### 2.2.2 回收率和精密度

基于优化的MDSPE样品前处理方法，在空白蜂蜜基质中进行3个水平（1、10、100 μg/kg）的加标回收试验（*n*=3）。如表6所示，3类目标物在低、中、高水平下的平均回收率如下：氯霉素85.2%~88.7%，喹诺酮类70.5%~109.3%，磺胺类70.5%~84.7%，相对标准偏差（RSD）<10.8%。由此可见，该方法在不同浓度梯度下均展现出稳定且出色的回收性能与精确度，满足蜂蜜中38种抗生素定量分析对方法可靠性与准确性的要求，适用于实际样品的检测分析。

### 2.3 实际样品测定

基于上述建立的MDSPE-HPLC-MS/MS方法，对42批次不同蜜源植物的蜂蜜样品进行38种抗生素残留检测，结果如图7所示。在所有蜂蜜样品中，共有19批次样品检测出至少1种抗生素残留，阳性检出率为45.2%。共检出12种抗生素，包括氯霉素、恩诺沙星、奥比沙星、加替沙星、马波沙星、诺氟沙星、氧氟沙星、司帕沙星、萘啶酸、磺胺嘧啶、磺胺间二甲氧嘧啶、磺胺喹喔啉。其中，氧氟沙星的阳性检出率最高，达21.4%，残留量范围为0.013~0.165 μg/kg，虽未超过推荐检测方法GB 31657.2-2021^［24］^的检出限，但这一现象提示今后需持续加强对其使用规范的技术指导，以防潜在的残留风险；诺氟沙星的检出水平最高，最高达1.61 μg/kg，另有2批次检出水平分别为0.75和0.56 μg/kg，均超过推荐检测方法的检出限但未超过国家标准GB 31650.1-2022^［25］^规定的最大残留限量（5.0 μg/kg），虽尚未造成超标风险，但其残留水平需引起关注，建议持续强化其在蜜蜂养殖中使用情况的监测和控制。值得注意的是，氯霉素在2批次中被检出，最高浓度为0.05 μg/kg，虽低于推荐检测方法GB/T 18932.19-2003^［26］^中0.1 μg/kg的检出限，但根据《农业农村部第250号公告》^［7］^，氯霉素已列为动物源食品中禁止使用的药物。此次检测结果表明，本研究方法可实现远低于现行限值的痕量定量能力，从而可更灵敏地筛查出禁用药物可能存在的非法使用问题。

**图7:**
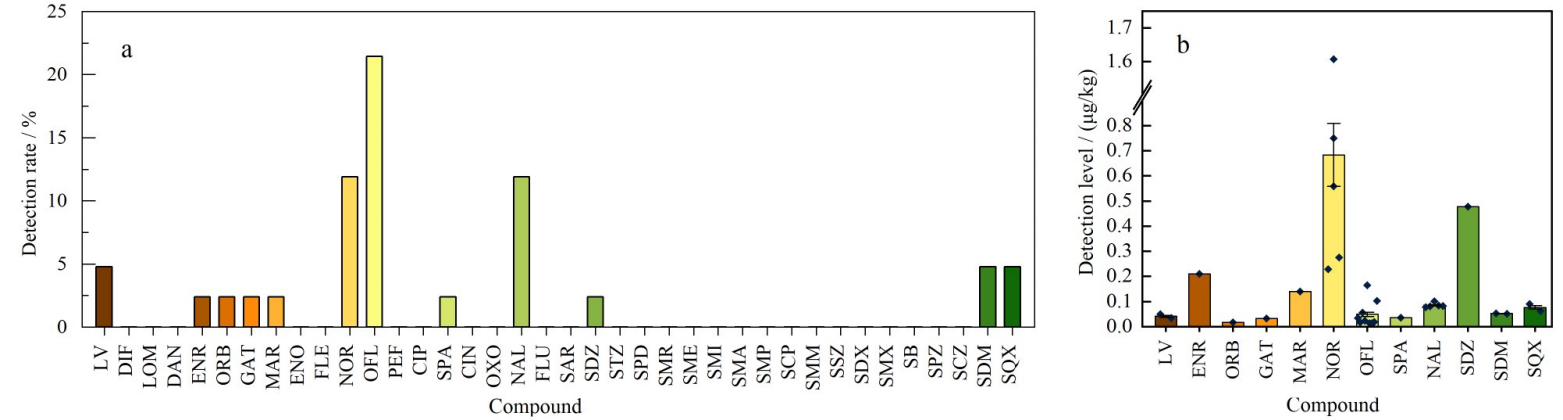
蜂蜜样品中38种抗生素残留的（a）检出率和（b）检出水平

此外，还检出了马波沙星、加替沙星等喹诺酮类药物以及磺胺嘧啶、磺胺喹噁啉、磺胺间二甲氧嘧啶等磺胺类药物，残留量均低于0.2 μg/kg。远低于国家标准GB 31650-2019^［27］^规定的磺胺类药物在食品动物中的最大残留限量100 μg/kg。

综上所述，本研究建立的MDSPE-HPLC-MS/MS检测方法具备高灵敏度和优异的筛查能力，可精准揭示蜂蜜中禁限用药物的潜在风险，凸显加强蜂产品中抗生素残留监测的必要性，有助于进一步完善蜂产品药物残留风险评估体系，保障蜂产品质量安全。

### 2.4 方法对比及绿色评估

为进一步评估蜂蜜中抗生素分析方法的性能表现及绿色化水平，本研究选取多种已报道的样品前处理策略进行比较，并基于AGREE对其绿色分析性能进行定量评估，如表7所示。Louppis等^［28］^开发了可用于检测蜂蜜中多种抗生素的LLE方法，但前处理过程包括离心、氮吹等多个耗时步骤，操作烦琐，时间和能耗成本较高。Varenina等^［29］^基于QuEChERS方法简化了操作流程，但仍消耗大量有机溶剂，绿色性欠佳。Goudarzi等^［30］^开发的SPE方法可同时提取蜂蜜基质中32种抗生素，但受所用萃取小柱的限制，方法灵敏度有待提高；近年来，MDSPE方法逐渐受到关注。Eyvazlou等^［31］^开发了一种磁性分散固相萃取方法，在操作便捷性和试剂绿色性方面具有优势，但其适用范围局限，仅覆盖4种磺胺类化合物，方法广谱性有限。相比之下，本研究开发的MDSPE方法在消耗少量样品的同时，大大简化了操作步骤，提取过程耗时短且无需大型设备，能够同步提取检测蜂蜜中多达38种抗生素，最低检出限可达0.01 μg/kg，绿色评分为0.73，在灵敏度、适用范围、绿色特性与分析效率等多个维度均展现出优异的性能（详见附表2）。

**表7:** 该方法与文献中蜂蜜中抗生素残留检测方法的对比

Compounds （quantity）	Pretreatment method	Determination/detection	LOD/（μg/kg）	LOQ/（μg/kg）	Recovery/%	AGREE assessment	Ref.
LV	LLE	LC-ESI-MS/MS	0.10	0.30	106.70	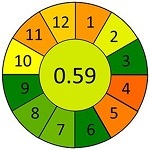	［^28^］
QNs （9）			3.60-5.30	10.80-14.70	90.10-115.80		
SAs （17）			1.90-9.20	9.90-27.60	77.40-105.60		
QNs （9）	QuEChERS	UHPLC-MS/MS	0.29-3.67	0.25-2.50	96.60-104.70	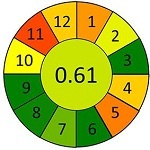	［^29^］
SAs （21）			0.13-3.26	0.10-2.50	94.20-103.40		
QNs （11）	SPE	LC-MS/MS	5.00	10.00	93.80-114.00	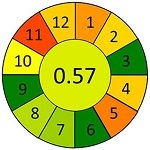	［^30^］
SAs （11）					94.40-110.30		
SAs （4）	MDSPE	HPLC-DAD	0.38-0.48	1.14-1.79	—	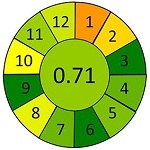	［^31^］
LV	MDSPE	HPLC-MS/MS	0.01	0.03	85.20-88.70	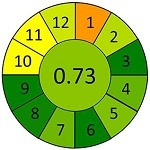	this work
QNs （17）			0.01-0.03	0.02-0.08	70.50-109.30		
SAs （18）			0.01-0.12	0.03-0.39	70.50-84.70		

## 3 结论

本研究基于mHLB磁性材料构建了一种磁分散固相萃取-高效液相色谱-串联质谱联用方法，可实现蜂蜜中氯霉素、喹诺酮类和磺胺类共38种抗生素的高效定量分析。该方法操作简便、提取效率高，检出限低，回收率良好，且具备优异的绿色性能，能够有效满足实际蜂蜜样品中痕量抗生素残留筛查的需求，可为蜂产品质量安全监测提供有力的技术支撑。此外，MDSPE有望与自动化磁性分离装置结合，实现样品制备流程的标准化与自动化，进一步提升检测效率与监测水平，推动蜂产品安全标准体系的完善，为精准监管与行业发展提供依据。
